# Screening mammography for second breast cancers in women with history of early-stage breast cancer: factors and causes associated with non-detection

**DOI:** 10.1186/s12880-018-0303-3

**Published:** 2019-01-05

**Authors:** Yoo Kyung Yeom, Eun Young Chae, Hak Hee Kim, Joo Hee Cha, Hee Jung Shin, Woo Jung Choi

**Affiliations:** Department of Radiology, Research Institute of Radiology, Asan Medical Center, University of Ulsan College of Medicine, 88, Olympic-ro 43-Gil, Songpa-Gu, Seoul, 05505 South Korea

**Keywords:** Breast cancer, Mammography, Screening, Second breast cancer

## Abstract

**Background:**

The aim of our study was to identify the factors and causes associated with non-detection for second breast cancers on screening mammography in women with a personal history of early-stage breast cancer.

**Methods:**

Between January 2000 and December 2008, 7976 women with early-stage breast cancer underwent breast surgery in our institution. The inclusion criteria of our study were patients who had: (a) subsequent in-breast recurrence, (b) surveillance mammography within 1 year before recurrence. Retrospective analysis of mammography was performed. Non-detection was defined as second breast cancers that were not visible on screening mammography. Imaging features, demographics, primary breast cancer (PBC) characteristics, and clinical features were evaluated to determine its association with non-detection. Univariate and multivariate logistic regression analyses were also performed to identify the factors related to non-detection.

**Results:**

We identified 188 patients that met the criteria. Among them, 39% of patients showed non-detection (*n* = 74). Of the 74 patients with non-detection, 53 (72%) were classified as having no detectable mammographic abnormality (i.e., true negative) due to overlapping dense breast tissue (*n* = 32), obscured by postoperative scar (*n* = 12) or difficult anatomic location / poor positioning (*n* = 9). The remaining 21 patients were categorized as having subtle findings (*n* = 11) or missed cancer (*n* = 10). Non-detection for second breast cancers were significantly associated with mammographic breast density (*p* = 0.001, OR = 2.959) and detectability of PBC on mammography (*p* = 0.011, OR = 3.013).

**Conclusion:**

Non-detection of second breast cancer in women with a personal history of early-stage breast cancer were associated with mammographic dense breast and lower detectability of PBC on mammography.

## Background

Breast cancer survival rates following diagnosis have improved through advances in local and systemic treatments and early detection [1], and early-stage breast cancers, including in situ (stage 0) and stage I-II, have a better prognosis than the later stages of invasive breast cancers [2]. At the same time, women with a personal history of early-stage breast cancer (PHBC) have an increased risk of second breast cancers [3], which can be either a local recurrence or a new primary cancer in the conserved and contralateral breast [4]. Studies have shown that early detection of second breast cancers in the asymptomatic phase has a better prognosis than patients with symptomatic disease [5]. A meta-analysis of 2263 breast cancer survivors showed that survival in both loco-regional and contralateral breast cancer recurrence was better in the early detection group (asymptomatic recurrence found by mammography) compared to women with symptomatic disease [6].

Surveillance of breast cancer patients after curative primary therapy focuses on the early detection of recurrent disease while still potentially curable. Current guidelines from the National Comprehensive Cancer Network and the American Society of Clinical Oncology recommend annual mammography for women with breast cancer following primary treatment [7–9]. In previous studies, 8 to 50% of ipsilateral recurrences and 18 to 80% of contralateral metachronous cancers were detected by mammography alone [3, 10]. This result highlights the need to identify causes and risk factors for non-detection on screening mammography for women with a PHBC. To our knowledge, only a few studies focused solely on interval cancer or false negative results have been published [11–14], but no studies have yet determined the reasons for non-detection that included all of them. Therefore, the purpose of this study was to identify the factors and causes associated with non-detection for second breast cancers on screening mammography in women with a PHBC.

## Methods

### Study population

Between January 2000 and December 2008, 7976 women (mean age, 48.9 years; age range, 18–88 years) with early-stage breast cancer, including ductal carcinoma in situ (DCIS) or stage I-II invasive carcinoma, underwent breast surgery in our institution. Our aim was to identify the factors and causes associated with non-detection for second breast cancers on screening mammography in women with a personal history of early-stage breast cancer. Therefore, the inclusion criteria of our study were women who had: (a) subsequent in-breast (ipsi- or contralateral) recurrence, and (b) surveillance mammography within 1 year before recurrence. We excluded patients whose mammography was not available in our picture archiving and communication system (*n* = 6). Institutional review board approval (No. 2017–0440) was obtained for this study, and the need for informed patient consent was waived owing to its retrospective nature.

### Definitions

In-breast recurrence included ipsilateral and contralateral recurrence. Ipsilateral recurrence was defined as local tumor recurrence in the same side after curative breast surgery. Metachronous breast cancer after primary cancer treatment in the opposite side was considered as contralateral recurrence.

Screening mammography after primary cancer treatment indicated to be a routine screen without any breast symptom. Non-detection was defined as second breast cancers that were not visible on screening mammography, including interval cancers (diagnosed before the next invitation to screening after negative screening mammography).

### Clinicopathologic data review

Clinicopathologic data were obtained from the electronic medical records. The clinical features of all patients were collected, including age at diagnosis of primary breast cancer (PBC), family history of breast cancer, menarcheal age, menopausal status, body mass index (BMI), breast feeding history, oral pill or hormonal therapy history, presence or absence of primary breast cancer symptoms, type of primary breast cancer surgery, and adjuvant treatment of primary breast cancer (radiotherapy, chemotherapy, and/or hormonal therapy).

The pathological data were also reviewed. The recorded data included pathologic tumor stage, histologic grade, lymph node (LN) status, lymphovascular invasion (LVI), and molecular subtype based on the expression of estrogen receptor (ER), progesterone receptor (PR), and human epidermal growth factor receptor 2 (HER2) status of both primary and recurrent tumors.

### Mammographic evaluation

All mammograms were obtained by using screen-film mammography units with a Senographe DMR scanner (GE Healthcare, Milwaukee, USA) or a Performa scanner (Instrumentarium), or dedicated digital mammography units with a Senographe DS or Senographe Essential unit (GE Healthcare). A combination of craniocaudal and mediolateral oblique views was evaluated.

All images were reviewed by two board-certified breast radiologists with ten and five years of clinical experience, respectively, and the final decision was carried out by consensus when a discrepancy occurred. Mammographic evaluation was done in two steps. The first step was blinded review. At this step, the radiologists were blinded to any clinical or histopathological information and other imaging results. They performed a retrospective review of the screening mammography whether the findings on screening mammography should be recalled or not. Cases that were not recalled by the blinded review were considered as non-detection.

The next step was unblinded review of mammography. In the case of non-detection from the first step of blinded review, the radiologists re-reviewed the mammography to determine the causes of non-detection. At that time, they were unblinded and used the other imaging results, such as ultrasound, magnetic resonance imaging, as available, to determine the reference location of the second breast cancers. The causes for non-detection of surveillance mammography were classified as ‘true negative’ or ‘interpretation error’. If the lesion could not be seen even in the unblinded repeat review, it was defined as ‘true negative’. And, the ‘interpretation error’ was subclassified as ‘missed’ or ‘subtle findings’. A cancer was defined as ‘missed’ if two radiologists agreed that the mammogram showed malignant signs that were overlooked or misdiagnosed in the first blinded review. If the mammogram showed nonspecific findings for malignancy, including asymmetry or a benign-appearing calcification, it was defined as ‘subtle finding’.

The visually estimated mammographic breast density was also determined for each patient based on the four categories of breast composition as described by the American College of Radiology (ACR) Breast Imaging Reporting and Data System (BI-RADS) [15]. Breast compositions ***c*** and ***d*** were classified as dense and breast compositions ***a*** and ***b*** were defined as fatty breast. The detectability of primary breast cancer on mammography was recorded from a review of the radiology reports during practice.

### Statistical analyses

Associations between the non-detection and categorical variables were assessed using chi-square or Fisher’s exact test, and t-test or Mann-Whitney test for continuous variables. Investigated factors included mammographic features (breast density and detectability of primary breast cancer on mammography), time interval between final diagnosis of secondary breast cancers and mammography, patient demographics, primary breast cancer tumor characteristics, and various clinical features. Univariate and multivariate logistic regression analyses were performed to identify the factors related to non-detection. A multivariable model using backward elimination was used to identify the independent factors associated with non-detection. The outputs were plotted, showing the adjusted odds ratio (OR) and the 95% confidence interval (CI) for each factor. All *p*-values < 0.05 were considered statistically significant. All statistical analyses were performed using SPSS version 12.0 (SPSS Inc., Chicago, IL, USA).

## Results

### Non-detection for second breast cancers

**Fig. 1:**
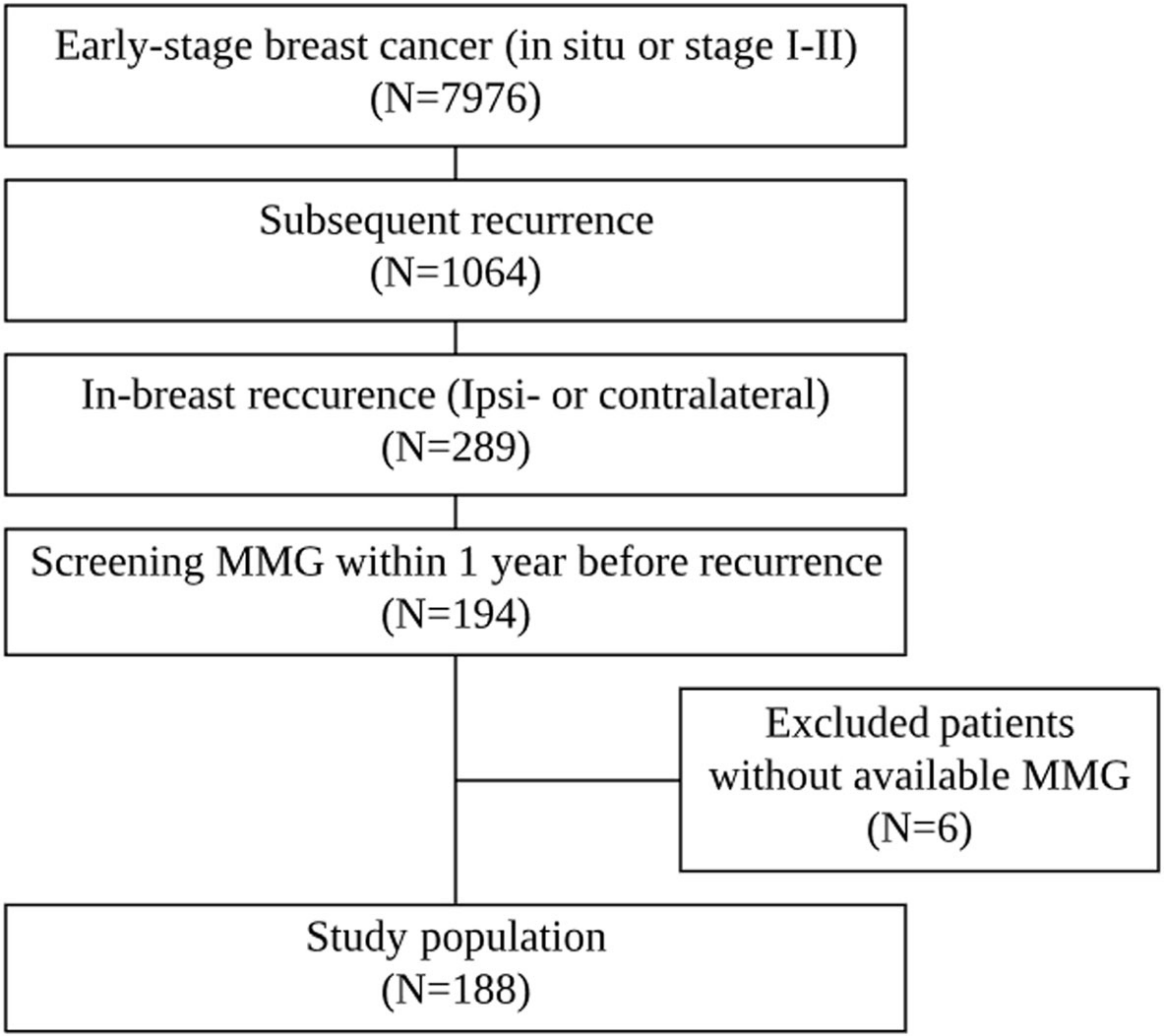
Flow chart of the study population

Among 7976 women with early-stage breast cancer who underwent breast surgery in our institution (Fig. 1), there were 1064 (13.3%) patients with subsequent recurrence. Of these, 289 patients were identified with in-breast recurrence and 194 patients had screening mammography within 1 year before recurrence. After excluding 6 patients whose mammography was unavailable, a total of 188 patients (mean age, 44 years; age range, 21–79 years) comprised our study population. The final diagnosis of the second breast cancers was based on surgery (*n* = 172), core needle biopsy (*n* = 13), or fine needle aspiration (*n* = 3). The mean time period from initial treatment to recurrence was 56 months (range, 5–152 months). Of those, 143 cases of ipsilateral recurrence and 48 cases of contralateral recurrence were identified. Recurrence in both sides was detected in 3 cases. Of the 188 patients, 86 (46%) second breast cancers showed the same molecular subtype as the primary breast cancer.

**Fig. 2:**
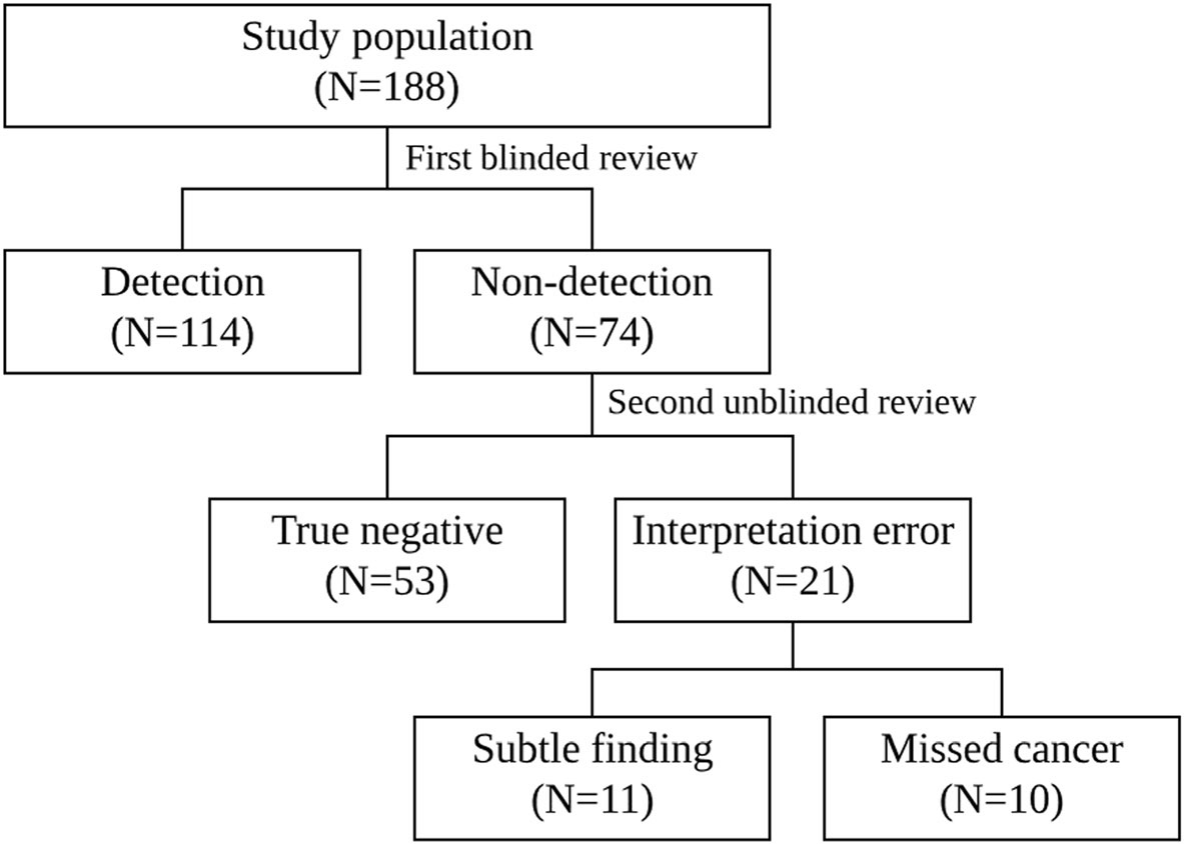
Two steps of mammographic evaluation

In the blinded mammographic review, 114 of 188 cases were detected by screening mammography (Fig. 2). In other words, 74 patients (39%) showed non-detection in the first blinded review.

### Causes associated with non-detection

**Fig. 3:**
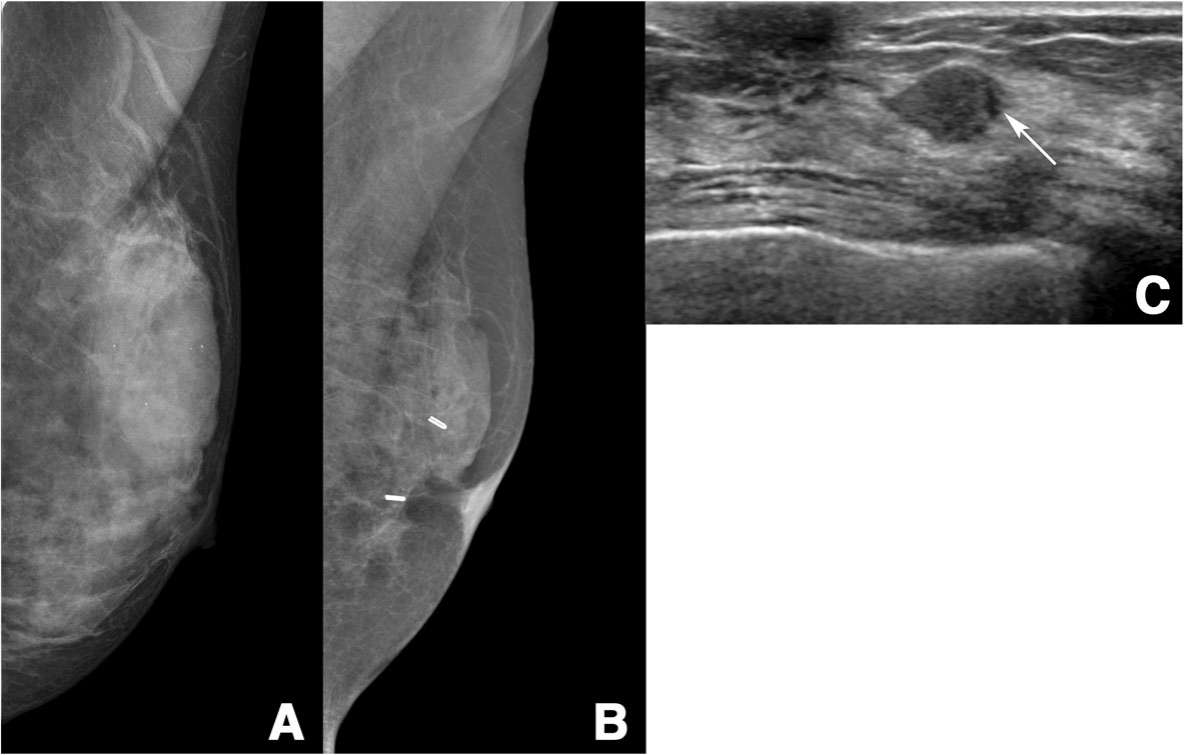
A 48-year-old woman with a PHBC in the left breast, classified as ‘true negative’. **a** Mediolateral oblique mammogram at primary breast cancer diagnosis showed extremely dense breast tissue and benign calcifications that were interpreted as negative. Primary breast cancer was a 22-mm microinvasive ductal carcinoma in the left breast. **b** Mediolateral oblique mammogram obtained 43 months after surgery also shows extremely dense breast tissue and no detectable abnormality except a postoperative change in the left breast. **c** Ultrasound image shows an 11-mm hypoechoic mass with partially not circumscribed margin (arrow) in the left breast subareolar area, which was pathologically proven to be a 12-mm microinvasive ductal carcinoma

**Fig. 4:**
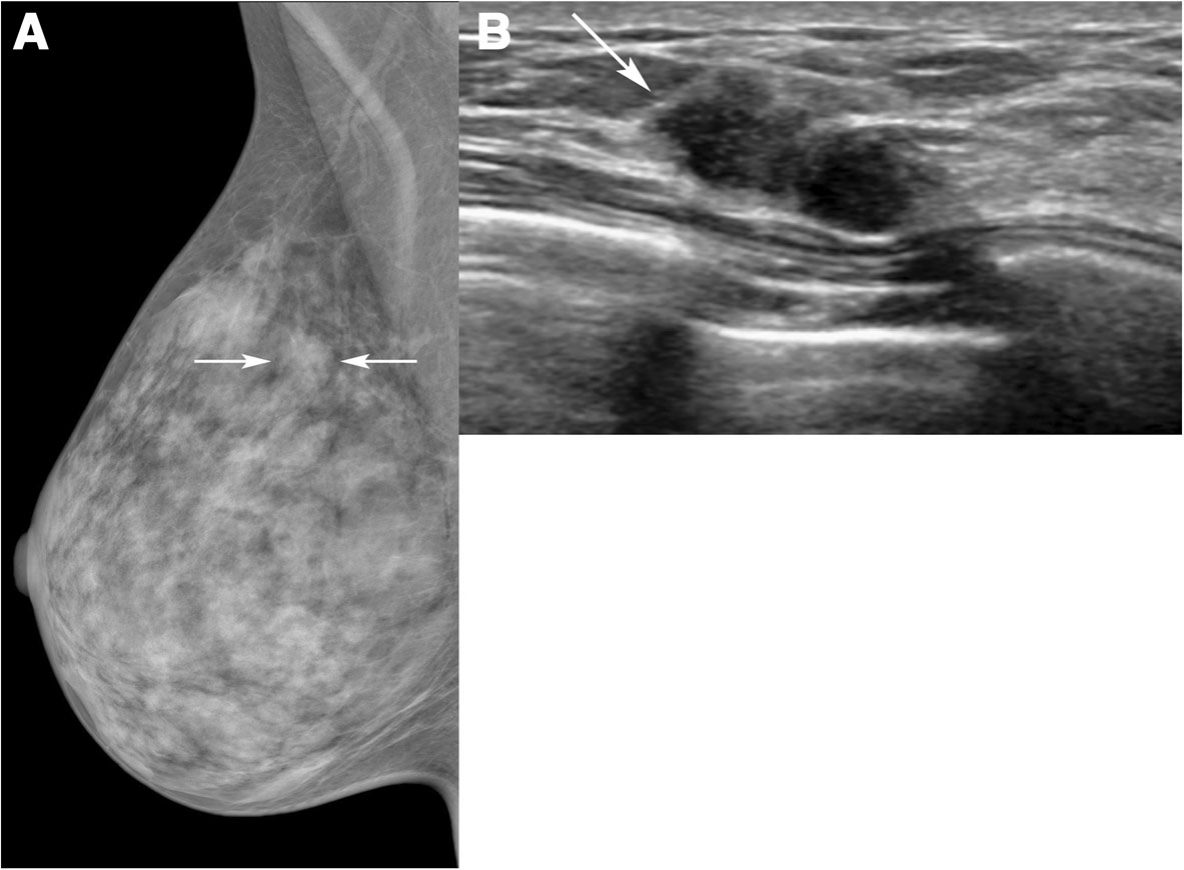
A 39-year-old woman with a PHBC in the left breast, classified as interpretation error, ‘subtle finding’. **a** Mediolateral oblique mammogram shows an asymmetry (arrows) on the right upper breast. **b** Ultrasound image shows a 15-mm irregular hypoechoic mass (arrow) in the right breast, which was pathologically proven to be an invasive ductal carcinoma

Of 74 patients with non-detection, 53 (72%) showed no detectable mammographic abnormality (i.e., ‘true negative’)(Fig. 3). In the unblinded repeat review using the reference location of the second breast cancers, 32 cases were obscured by overlapping dense breast tissue, 12 cases were obscured by postoperative scar, and 9 cases were not included due to difficult anatomic location or poor positioning. The remaining 21 patients were classified as ‘interpretation error’, including 11 subtle findings and 10 missed cancers. Subtle findings (Fig. 4) included asymmetry (*n* = 8) or benign-appearing calcification (*n* = 3), and ‘missed cancer’ showed a mass (*n* = 6), calcification (*n* = 3), or focal asymmetry (*n* = 1).

### Factors associated with non-detection

Tables 1 and 2 summarize the association between clinical features, tumor characteristics, and non-detection. The clinical features were not significantly different between those with or without non-detection, except for family history (*p* = 0.043). Also, no significant difference was observed between these two groups in terms of primary breast cancer tumor characteristics.

**Table 1:** Association between clinical features and non-detection

Variables		Non-detection (*n* = 114)	Detected (*n* = 74)	*P* value
Age at PBC diagnosis, years (mean ± SD)		44.6 ± 11.5	43.0 ± 11.2	0.352
Menarcheal age, years (mean ± SD)		14.5 ± 1.6	14.1 ± 1.6	0.105
Menopausal status	Pre	84 (58%)	60 (42%)	0.242
	Post	30 (68%)	14 (32%)	
Body mass index	< 25	91 (61%)	59 (39%)	0.987
	≥ 25	23 (61%)	15 (39%)	
Type of surgery	Conservation	92 (60%)	62 (40%)	0.592
	Mastectomy	22 (65%)	12 (35%)	
Symptom of PBC*	Yes	79 (58%)	58 (42%)	0.171
Breast feeding history	Yes	64 (60%)	42 (40%)	0.934
Oral pill history	Yes	10 (56%)	8 (44%)	0.642
Hormonal therapy	Yes	11 (85%)	2 (15%)	0.067
Family history	Yes	17 (81%)	4 (19%)	0.043
Adjuvant radiotherapy	Yes	82 (59%)	58 (41%)	0.322
Adjuvant chemotherapy	Yes	57 (56%)	44 (44%)	0.204
Adjuvant hormonal therapy	Yes	56 (57%)	42 (43%)	0.306

Note—The numbers in parentheses are percentages

*PBC primary breast cancer

**Table 2:** Association between tumor characteristics and non-detection

Variables		Non-detection (*n* = 114)	Detected (*n* = 74)	*P* value
Recurrence site	Ipsilateral	85 (61%)	55 (39%)	1.000
	Contralateral	27 (60%)	18 (40%)	
	Both	2 (67%)	1 (33%)	
Pathologic stage	Stage 0	26 (67%)	13 (33%)	0.357
	Stage 1	53 (63%)	31 (37%)	
	Stage 2	35 (54%)	30 (46%)	
Histologic grade	Grade 1	13 (81%)	3 (19%)	0.249
	Grade 2	60 (62%)	37 (38%)	
	Grade 3	37 (55%)	30 (45%)	
	Unknown	4 (50%)	4 (50%)	
LN positivity	Yes	14 (50%)	14 (50%)	0.212
Lymphovascular invasion	Yes	18 (62%)	11 (38%)	0.864
ER	Positive	61 (64%)	35 (36%)	0.405
PR	Positive	61 (65%)	33 (35%)	0.232
HER2	Negative	74 (57%)	55 (43%)	0.161
	Positive	36 (65%)	19 (35%)	
	Equivocal	4 (100%)	0 (0%)	
Molecular subtype	Luminal A	51 (61%)	32 (39%)	0.211
	Luminal B	17 (71%)	7 (29%)	
	HER2	19 (61%)	12 (39%)	
	TN*	23 (50%)	23 (50%)	
	Unknown	4 (100%)	0 (0%)	

Note—The numbers in parentheses are percentages

*TN triple negative

Table 3 shows the association between mammographic features and non-detection. Mammographic breast density was significantly associated with non-detection (*p* < 0.001). Non-detection for second breast cancers was significantly more frequent when primary breast cancer was not detectable on mammography compared to when it was detectable (*p* = 0.007). There was no statistically significant difference in the time interval between the final diagnosis of second breast cancers and mammography.

**Table 3:** Association between mammographic features and non-detection

Variables		Non-detection (*n* = 114)	Detected (*n* = 74)	*P* value
Mammographic breast density	Fatty	71 (74%)	25 (26%)	< 0.001
	Dense	43 (47%)	49 (53%)	
Mammographic detectability of PBC	Positive	85 (66%)	44 (34%)	0.007
	Negative	11 (35%)	20 (65%)	
	NA	18 (64%)	10 (36%)	
Time interval (days)^a^,^b^		32.5 (15–60)	39.0 (12–160)	0.306

*NA* not available

^a^Data represent the median (interquartile range)

^b^Time interval between the final diagnosis of second breast cancers and mammography

In the multivariate analysis, mammographic breast density and detectability of primary breast cancer on mammography, remained independent variables related to non-detection (Table 4). The odds ratios were 2.959 (95% CI: 1.581, 5.540) for breast density and 3.013 (95% CI: 1.290, 7.041) for mammographic detectability of primary breast cancer.

**Table 4:** Univariate and multivariate analysis of factors associated with non-detection

Variables		Univariate			Multivariate		
		Odds ratio	95% CI	*P value*	Odds ratio	95% CI	*P value*
Mammographic breast density	Fatty	1		< 0.001	1		0.001
	Dense	3.236	1.754–5.973		2.959	1.581–5.540	
Mammographic detectability of PBC	Positive	1			1		
	Negative	3.512	1.546–7.982	0.003	3.013	1.290–7.041	0.011
	NA	1.073	0.457–2.522	0.871	1.172	0.484–2.840	0.725

*NA* not available

## Discussion

As breast cancer survivors are at a higher risk for second breast cancer in the conserved and opposite breasts, women with breast cancer are recommended for regular imaging surveillance after primary treatment. Our study of the screening mammography in women with a personal history of early-stage breast cancer revealed that 39% of patients showed non-detection for second breast cancer. Our study also shows that non-detection for second breast cancer was associated with mammographic breast density and the detectability of primary breast cancer on mammography.

In our study, the most common cause of non-detection by screening mammography was ‘true negative’ cancer. Fifty-three (72%) of 74 patients with non-detection showed no detectable mammographic abnormality in the unblinded repeat review with the reference location of the second breast cancers. 32 (43%) cases were obscured by overlapping normal breast tissue and 12 (16%) cases were obscured by postoperative scar. Breast cancer can be invisible on mammography if the tumor does not show a higher density distinct from surrounding fibroglandular tissue or postoperative scar. The remaining 9 (12%) cases of ‘true negative’ cancer were not included due to difficult anatomic location or poor positioning. These cancers more frequently were located at the immediate prepectoral region. Schrading and Kuhl [16] reported that the posterior location was observed more frequently in women at high risk for breast cancer and women with BRCA mutations. Thus, they suggested breast positioning during mammographic examinations should be optimized to include the posterior tissues, particularly in women at high risk with dense breasts.

The remaining 21 (28%) non-detection patients were classified as ‘interpretation error’, including 11 subtle findings and 10 missed cancers. The percentage of interpretation error in our study was similar to previous results from the ACRIN 6666 study and other reports on mammographically missed cancers [17–19]. In the ACRIN study, a retrospective review revealed that 19 of 67 (28%) mammographically undetected cancers were interpretation errors. It meant that although the lesions were visible on mammography, they were considered as probably benign findings, such as asymmetry or benign-appearing calcification. Of the 21 misinterpreted cases at mammography in our study, the lesions were a mass or asymmetry in 15 cases and calcification in 6 cases. These findings are consistent with those of previous studies that reported that noncalcified findings were more common than calcifications among interpretation errors [20, 21].

Our multivariate analysis found that mammographic breast density (OR = 2.959; *p* = 0.001) and mammographic detectability of primary breast cancer (PBC; OR = 3.013; *p* = 0.011) are independent variables associated with non-detection for second breast cancers. Mammographic breast density is one of the known risk factors for breast cancer and it also makes the detection of cancer by mammography more difficult [22]. Thus, breast density is a major issue in breast cancer screening because it is one of the variables proposed for tailored screening [23, 24]. In an analysis of missed cancers at screening mammography, Bird et al. reported that missed cancers occurred in women with denser breasts. These findings are similar with those in our study of postoperative patients, which showed that 66% of patients with non-detection had dense breast tissues and 62% of patients without non-detection had fatty breasts by mammography [25].

We also identified that the detectability of primary breast cancer on mammography is associated with non-detection for second breast cancers. Yang et al. [26] reported that clinical and pathologic differences in mammographically occult and mammographically positive primary tumors ultimately result in more false-negative mammograms at recurrence for the mammographically occult cohort. The results of the Yang et al. study showed lower mammographic detectability of the recurrent cancer in the mammographic occult cohort after breast-conserving operation. Breast density is influenced by age, parity, body mass index, and menopause, but these factors account for only 20–30% of the variation in breast density in the population. Twin studies have shown that the percent of mammographic density is highly heritable [22]. Furthermore, a significant portion (46%) of second breast cancers in our study showed the same molecular subtype as the primary breast cancer. This result is similar to those in previous studies, which reported that 48.3–71.9% of recurrent breast cancers had no changes in hormone receptors and HER2 status from the primary breast cancer [27–29].

Currently, there is a lack of consistent data on appropriate screening strategies to potentially reduce non-detection of breast cancers in women with a PHBC participating in mammography screening. The addition of ultrasonography or MRI may have its strong points for visualizing areas that cannot be approached by mammography and could provide additional information regarding differentiation between postoperative changes and tumor recurrence, especially in high risk women [30]. Recently, Cho et al. showed that addition of MRI to mammography screening improved the detection of early-stage breast cancers in women treated with breast conserving therapy [4]. It is worthwhile to know which group of patients may benefit more from the adjunct screening in women with a PHBC. Our study revealed that mammographic dense breast and lower detectability of primary breast cancer on mammography are associated with non-detection for second breast cancer in women with a PHBC. These findings can provide indirect evidence that adjunct screening tools may be necessary for these patients.

Our study had limitations. First, this was a retrospective study conducted at a single institution, although we did include quite a large number of consecutive patients. Second, not all of the mammograms were obtained by digital mammography, which may have affected the outcome. Thus, prospective multi-institutional evaluation is needed to eliminate bias based on differences that may exist in patient demographics, diagnostic equipment, or therapeutic interventions.

## Conclusion

Our study revealed that the non-detection of second breast cancer in women with a personal history of early-stage breast cancer were associated with mammographic dense breast tissue and lower detectability of primary breast cancer on mammography. Most breast cancers with non-detection were not identified by mammography, even in an unblinded review. Further study is necessary to evaluate the role of adjunct screening tools in patients with a history of early-stage breast cancer.

## Abbreviations

BI-RADS: Breast imaging reporting and data system
DCIS: Ductal carcinoma in situ
PBC: Primary breast cancer
PHBC: Personal history of breast cancer
